# Improvement of ALT decay kinetics by all-oral HCV treatment: Role of NS5A inhibitors and differences with IFN-based regimens

**DOI:** 10.1371/journal.pone.0177352

**Published:** 2017-05-18

**Authors:** Valeria Cento, Thi Huyen Tram Nguyen, Domenico Di Carlo, Elisa Biliotti, Laura Gianserra, Ilaria Lenci, Daniele Di Paolo, Vincenza Calvaruso, Elisabetta Teti, Maddalena Cerrone, Dante Romagnoli, Michela Melis, Elena Danieli, Barbara Menzaghi, Ennio Polilli, Massimo Siciliano, Laura Ambra Nicolini, Antonio Di Biagio, Carlo Federico Magni, Matteo Bolis, Francesco Paolo Antonucci, Velia Chiara Di Maio, Roberta Alfieri, Loredana Sarmati, Paolo Casalino, Sergio Bernardini, Valeria Micheli, Giuliano Rizzardini, Giustino Parruti, Tiziana Quirino, Massimo Puoti, Sergio Babudieri, Antonella D’Arminio Monforte, Massimo Andreoni, Antonio Craxì, Mario Angelico, Caterina Pasquazzi, Gloria Taliani, Jeremie Guedj, Carlo Federico Perno, Francesca Ceccherini-Silberstein

**Affiliations:** 1 Department of Experimental Medicine and Surgery, University of Rome Tor Vergata, Rome, Italy; 2 INSERM, Université Paris Diderot, IAME, UMR 1137, Sorbonne Paris Cité, Paris, France; 3 Tropical Diseases, Umberto I Hospital – “Sapienza” University, Rome, Italy; 4 Infectious Diseases, Sant’Andrea Hospital – “Sapienza” University, Rome, Italy; 5 Hepatology Unit, Polyclinic of Rome Tor Vergata, Rome, Italy; 6 Gastroenterology, “P. Giaccone” University Hospital, Palermo, Italy; 7 Infectious Diseases, Polyclinic of Rome Tor Vergata, Rome, Italy; 8 Clinic of Infectious Disease, Department of Health Sciences, San Paolo University Hospital, University of Milan, Milan, Italy; 9 Department of Biomedical, Metabolic and Neural Sciences, NOCSAE Baggiovara, Baggiovara, Modena, Italy; 10 Infectious Diseases Unit, Department of Clinical and Experimental Medicine, University of Sassari, Sassari, Italy; 11 Infectious Diseases, AO Ospedale Niguarda Cà Granda, Milan, Italy; 12 Infectious Diseases, Ospedale di circolo di Busto Arsizio, Busto Arsizio, Varese, Italy; 13 Infectious Disease Unit, “Spirito Santo” General Hospital, Pescara, Italy; 14 Gastroenterology, Catholic University of Rome, Rome, Italy; 15 University of Genoa (DISSAL) Infectious Diseases Unit/AOU IRCCS San Martino-IST, Genoa, Italy; 16 1^st^ Division of Infectious Diseases, ASST Fatebenefratelli Sacco, Milan, Italy; 17 Istituto Nazionale di Genetica Molecolare (INGM) "Romeo ed Enrica Invernizzi", Milan, Italy; 18 Clinical Microbiology, Virology and Bioemergencies, ASST Fatebenefratelli Sacco, Milan, Italy; 19 School of Clinical Medicine, Faculty of Health Science, University of the Witwatersrand, Johannesburg, South Africa; Kaohsiung Medical University Chung Ho Memorial Hospital, TAIWAN

## Abstract

**Background:**

Intracellular HCV-RNA reduction is a proposed mechanism of action of direct-acting antivirals (DAAs), alternative to hepatocytes elimination by pegylated-interferon plus ribavirin (PR). We modeled ALT and HCV-RNA kinetics in cirrhotic patients treated with currently-used all-DAA combinations to evaluate their mode of action and cytotoxicity compared with telaprevir (TVR)+PR.

**Study design:**

Mathematical modeling of ALT and HCV-RNA kinetics was performed in 111 HCV-1 cirrhotic patients, 81 treated with all-DAA regimens and 30 with TVR+PR. Kinetic-models and Cox-analysis were used to assess determinants of ALT-decay and normalization.

**Results:**

HCV-RNA kinetics was biphasic, reflecting a mean effectiveness in blocking viral production >99.8%. The first-phase of viral-decline was faster in patients receiving NS5A-inhibitors compared to TVR+PR or sofosbuvir+simeprevir (p<0.001), reflecting higher efficacy in blocking assembly/secretion. The second-phase, noted δ and attributed to infected-cell loss, was faster in patients receiving TVR+PR or sofosbuvir+simeprevir compared to NS5A-inhibitors (0.27 vs 0.21 d^-1^, respectively, p = 0.0012). In contrast the rate of ALT-normalization, noted λ, was slower in patients receiving TVR+PR or sofosbuvir+simeprevir compared to NS5A-inhibitors (0.17 vs 0.27 d^-1^_,_ respectively, p<0.001). There was no significant association between the second-phase of viral-decline and ALT normalization rate and, for a given level of viral reduction, ALT-normalization was more profound in patients receiving DAA, and NS5A in particular, than TVR+PR.

**Conclusions:**

Our data support a process of HCV-clearance by all-DAA regimens potentiated by NS5A-inhibitor, and less relying upon hepatocyte death than IFN-containing regimens. This may underline a process of “cell-cure” by DAAs, leading to a fast improvement of liver homeostasis.

## Introduction

In 1998 Neumann and his colleagues proposed a seminal model to explain the biphasic decline of HCV-RNA after initiation of interferon (IFN)[1]. According to this model, the rapid viral-load decline in the first days of treatment depends upon virus clearance from plasma, with a magnitude reflecting the treatment effectiveness in blocking viral production. After this rapid “first-phase”, a slower”second-phase” ensues, consistent with progressive elimination of infected hepatocytes.

In the last 20 years, this initial model was expanded to include novel ideas[2–5], and novel drugs. One important point raised by scientists was that the second-phase of viral kinetics during direct-acting antivirals (DAAs) treatment may not only represent cell-elimination, but may also be associated with the progressive reduction of the intracellular viral content, down to its disappearance (i.e. “cell-cure”)[6–8]. This possibility was supported by experiments *in vitro*[9] and by the clinical observation that the second-phase with DAAs was faster than during IFN-based therapy, and no increase in Alanine Amino Transferases (ALT) levels was observed[7, 10].

ALT levels can be considered as a surrogate marker for cell-death, since hepatic injury causes their leak from damaged hepatocytes and their increase in serum[11]. Since IFN-activity includes cell-death, it was not uncommon to observed suboptimal ALT decline, and infrequent ALT-normalization, during IFN-treatment, especially in patients with more compromised liver status[12].

Consistently with the idea of a “cell-cure” by DAAs, ALT kinetics is thus supposed to be improved. Telaprevir (TVR) indeed allowed ALT-normalization in 62% of patients by day-9 when used in monotherapy (vs. 33% when used with pegIFN and ribavirin [PR])[13], and the IFN-free association of SOF+ledipasvir+GS9669/GS-9451 allowed ALT reduction to normal levels by day-14 in 90% of patients[14]. Nevertheless, no data are available on ALT kinetics with currently-used DAA combinations in clinics, nor on the impact of cirrhosis, making difficult to appreciate the role of all-DAA combinations in the more complex patients seen in real-world practice.

Here, in order to better understand the effect of DAA combinations, we compared the kinetics of both ALT and HCV-RNA in cirrhotic-patients treated with various DAA-based regimens, with or without IFN, for whom frequent measurements of both markers were available. We used the techniques of mathematical modeling to recapitulate the kinetics observed and better understand the origin of possible differences across treatment-regimens.

## Materials and methods

### Study population

Adults aged ≥18 years with genotype-1 infection and cirrhosis, receiving one DAAs with or without IFN were included if had: a) baseline and week-4 HCV-RNA and ALT available; b) at least one HCV-RNA determination within the first 6 days of treatment, and one HCV-RNA and ALT determination at week-1 and/or at week-2; c) high baseline ALT (>55 IU/ml in men; >45 IU/ml in women). Patients who prematurely ended treatment before week-4 were excluded.

Cirrhosis was documented by liver-biopsy or Fibroscan (≥12.5 KPa).

Detailed information on sampling and laboratory analyses are reported in S1 Supplementary Material.

Approval by the local Ethics Committees of Fondazione PTV Policlinico Tor Vergata and patient written informed consent were obtained. This study was conducted in accordance with the principles of the Declaration of Helsinki. All information were recorded in an anonymous database.

### Statistical analysis

Results were compared using Spearman's rank correlation test, Mann-Whitney U-test, Fisher exact test or Chi^2^ test.

Cox regression analysis was performed using the following confounding variables: IFN-administration, HCV-subtype, baseline ALT, NS5A-inhibitors administration, HCV-RNA decay baseline-2 weeks, baseline HCV-RNA, previous IFN-experience (relapse, no-response, other), previous PI-failure, IL-28B CC genotype, and ribavirin co-administration. Only variables having a p-value <0.10 in univariate analysis were included in multivariable analysis. To avoid statistical multicollinearity, IFN and NS5A-administration were analyzed separately. Statistical significance threshold was set at 0.050.

All statistical analyses were performed using the open source environment R and SPSS software package (SPSS Inc., Chicago, Illinois).

### Mathematical modeling of early HCV-RNA and ALT kinetics

As we focused on early HCV-RNA and ALT kinetics, only data obtained during the first 4 weeks of treatment were analyzed. Data below LLOQ were taken into account using the extended SAEM algorithm implemented in MONOLIX 4.3.3.

HCV-RNA and ALT kinetics were analyzed separately using nonlinear mixed-effect models, optimized according to significant covariates as described in S1 Supplementary Material.

To illustrate the difference in the virological and biological response across the covariates, we simulated 1000 individual profiles from the final parameter estimates.

On the best model for viral and ALT kinetics containing only significant covariates, the correlation between viral and ALT kinetic parameters, stratified or not on the different treatment groups, was studied using Spearman correlation test. A joint model that would simultaneously fit HCV-RNA and ALT kinetic was considered relevant if at least one correlation coefficient was >0.4.

## Results

### Characteristics of the study population

111 patients met the inclusion criteria (Table 1), of whom 81 treated with an all-DAAs regimen: 32 with paritaprevir/r+ombitasvir+dasabuvir (3D); 9 with daclatasvir+asunaprevir (DCV+ASU); 5 with DCV+SOF; and 35 with simeprevir (SIM)+SOF. In addition, 30 patients received TVR+PR. Ribavirin was co-administered in all patients treated with 3D, in 60% of patients treated with DCV+SOF or SOF+SIM.

**Table 1:** Characteristics of the study population.

		IFN-free, all-DAA regimens				Telaprevir and pegIFN	P-value^a^
		Paritaprevir, ombitasvir and dasabuvir	Daclatasvir and asunaprevir	Daclatasvir and sofosbuvir	Sofosbuvir and simeprevir		
**Patients, N**		32	9	5	35	30	
**HCV genotype, N(%)**	1a	8 (25.0)	0 (0.0)	2 (40.0)	20 (57.1)	13 (43.3)	0.545
	1b	24 (75.0)	9 (100)	3 (60.0)	15 (42.9)	17 (56.7)	
**Males, N(%)**		23 (71.9)	6 (66.7)	4 (80.0)	24 (68.6)	22 (73.3)	0.760
**Age (years), Median (IQR)**		58 (53–65)	65 (56–68)	53 (50–54)	56 (51–64)	55 (51–63)	0.300
**Stiffness at baseline (Kpa), Median (IQR)**^b^		26.7 (17.3–33.8)	28.4 (16–31)	22.8 (12.1–72)	20.4 (16.6–27.7)	21 (19–26)	0.367
**Naive patients, N(%)**		6 (18.8)	1 (11.1)	1 (20.0)	8 (22.9)	7 (23.3)	0.793
**Treatment experience, N(%)**	Non responder	17 (53.1)	6 (66.7)	2 (40.0)	17 (48.6)	15 (50)	1.000
	Relapser	7 (21.9)	2 (22.2)	2 (40.0)	5 (14.3)	8 (26.7)	0.445
	Breakthrough	0 (0.0)	0 (0.0)	0 (0.0)	2 (5.7)	0 (0.0)	1.000
	Other	2 (6.3)	0 (0.0)	0 (0.0)	3 (8.6)	0 (0.0)	0.321
**PI experienced, N(%)**		2 (6.3)	0 (0.0)	1 (20.0)	5 (14.3)	0 (0.0)	0.074
**RBV administration, N(%)**		32 (100)	0 (0.0)	3 (60.0)	21 (60.0)	30 (100)	0.001
**Baseline HCV-RNA (logIU/ml), Median (IQR)**		5.5 (5.1–6)	5.8 (5.4–6.1)	5.5 (5.3–6.3)	5.8 (5.2–6.2)	6.0 (5.7–6.7)	0.006
**Baseline ALT (IU/ml), Median (IQR)**		74 (60–127)	118 (70–155)	111 (102–139)	96 (77–166)	95 (65–144)	0.963
**Baseline AST (IU/ml), Median (IQR)**		85 (63–125)	133 (91–170)	128 (122–134)	96 (72–129)	72 (51–100)	0.015
**SVR**_**12**_**, N (%)**		32 (100)	8 (88.9)	4 (80.0)	32 (91.4)	20 (66.7)	<0.001

^a^ P-values were calculated by Fisher exact test for categorical variables and by Mann-Whitney test for continuous variables. The comparison was performed for telaprevir and pegIFN patients *vs*. all DAA-treated patients.

^b^ Baseline stiffness value was available for 30 paritaprevir, ombitasvir and dasabuvir treated patients, 9 daclatasvir and asunaprevir patients, 3 daclatasvir and sofosbuvir patients, 29 sofosbuvir and simeprevir patients, and 21 telaprevir and pegIFN treated patients.

ALT, alanine transaminase; AST, aspartate aminotransferase; DAAs, Direct Acting Antivirals; HCV, hepatitis C virus; IFN, interferon; IQR, interquartile range; PI, protease inhibitor; RBV, ribavirin; SVR_12_, sustained virological response after 12 weeks of follow-up.

**Fig 1:**
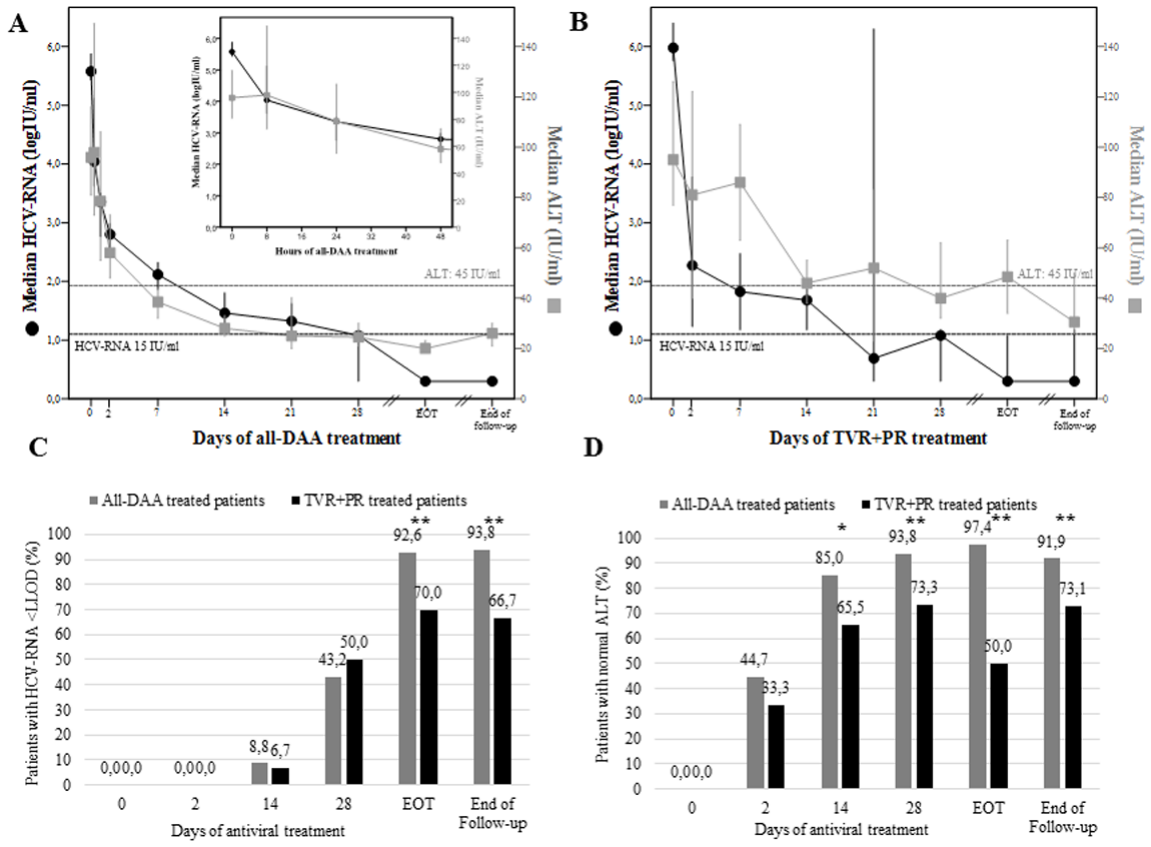
Biphasic kinetics of HCV-RNA decay, and ALT drop during all-DAA and TVR+PR treatment and follow-up. In *upper panels*, median values with 95% confidence interval of HCV-RNA (black dots) and ALT (grey squares) during all-DAAs (panel **A**) and TVR+PR (panel **B**) treatment are reported. End of follow-up is at 12 weeks after treatment discontinuation. Black dotted line represents the lower limit of detection of HCV-RNA (12–15 IU/ml). Grey dotted line represents normality range of ALT values in females (45 IU/ml). Histograms in *lower panels* represent the percentages of patients with HCV-RNA below the lower limit of detection (panel **C**) and with normal ALT values (panel **D**) during all-DAAs (black) and TVR+PR (grey) treatment. Normal ALT values were considered as <55 IU/ml in men, and <45 IU/ml in women. ALT, alanine transaminase; DAA, direct-acting antivirals; EOT, end of treatment; IU, international units; LLOD, lower limit of detection (<12–15 IU/ml, not detected); PR, pegylated interferon and ribavirin; TVR, telaprevir. * p-value <0.05 by Fisher exact test; ** p-value ≤0.001 by Fisher exact test.

A sustained virological response (SVR_12_) was achieved in 76/81 (93.8%) all-DAA patients and in 20/30 TVR+PR patients (66.7%, p<0.001) (Fig 1 panel A). Among TVR+PR patients, 9 discontinued treatment prematurely for virological reasons: 3 according to stopping-rules for partial-response, and 6 for virological-breakthrough. Overall, 6 patients relapsed: 1 treated with DCV+ASU, 1 with DCV+SOF, 3 with SOF+SIM and 1 with TVR+PR. Six patients ended all-DAA treatment with HCV-RNA detectable but not quantifiable, and were eventually SVR_12_.

All-DAA and TVR+PR populations had comparable median (IQR) baseline ALT values (96 [9–138] *vs*. 95 [65–144], respectively; p = 0.963), and liver stiffness (23.6 [16.5–31.6] *vs*. 21.0 [19.0–26.0] kPA, respectively; p = 0.367). At least 1 baseline RAS (in relation to the actual regimen) was found in 22/91 (24.2%) all-oral treated patients, of whom 17 were infected with GT-1b and 5 with GT-1a.

### Patterns of HCV-RNA and ALT decay during and after antiviral treatment

In the first 4 weeks of treatment, a fast first-phase HCV-RNA decline in serum, followed by a slower second-phase, was observed in all 111 patients analyzed (Fig 1, panels A and B). No significant differences were found in HCV-RNA response rates among all-DAA and TVR+PR treated patients, at least until day-28 (Fig 1, panel C). On the contrary, and despite comparable baseline ALT values, TVR+PR patients showed a more heterogeneous, and generally suboptimal ALT decline compared to all-DAA patients (Fig 1, panels B). Indeed, all-DAAs treatment led to ALT normalization in 85.0% of patients after only 14 days, and in 93.8% after 28 days, while only 65.5% of TVR+PR patients had normal ALT at day-14 (p = 0.02), and 73.3% at day-28 (p = 0.001). The achievement of healthy ALT during treatment required a median (IQR) of 7 (7–14) days for all-DAA patients, vs. 14 (14–39) in TVR+PR patients, even though this estimation can be affected by different sampling times.

Between day-28 and the end of treatment (EOT), 7/28 (25%) TVR+PR patients showed a re-alteration of ALT values after normalization, *vs*. 1/77 (1.3%) all-DAA patients (p<0.001) (data not shown). At EOT, 97.4% of all-DAA patients had normal ALT values, vs. only 50.0% of TVR+PR patients (p<0.001).

After 12 weeks since treatment discontinuation, the proportion of patients with normal ALT values among those who had received IFN increased form 50% to 73.1%, vs. 91.9% of all-DAA patients (p<0.001) (Fig 1, panel D). At the last follow-up available after SVR (median [IQR] since treatment end = 91 [84–165] days), 65/69 all-DAA and all 13 TVR+PR patients had healthy ALT.

ALT normalization rates at EOT were not significantly affected by baseline RASs presence (p = 0.510), nor were SVR rates (p = 0.386), not even in patients with baseline HCV-RNA > 800.000 IU/ml (p = 0.152).

### Impact of different DAA-combinations on modeled HCV-RNA kinetics

The kinetics of HCV-RNA decline was well described by a standard biphasic model, whose population-parameters are reported in Table 2.

**Table 2:** Population kinetic parameters for the best HCV and ALT kinetic model.

Parameters^a^		Fixed effect (RSE%)	p-value	Standard deviation (RSE%)
**HCV-RNA Biphasic model**				
**VL**_**0**_ (logIU/ml)	GT 1a	5.98 (2)	0.00086	0.099 (7)
	GT 1b	5.61 (1)		
**c** **(/day)**	NS5A-containing	7.88 (8)	0.00071	0.34 (15)
	SOF+SMV and TVR+PR	5.28 (9)		
**δ** **(/day)**	NS5A-containing	0.21 (6)	0.0012	0.415 (8)
	NS5A-free	0.27 (5)		
**ε**	All-DAA	0.998 (2)	0.01	0.16 (10)
	TVR+PR	0.999 (4)		
**Additive residual errors**				0.288 (4)
**ALT Monophasic model**				
**ALT**_**0**_ **(IU/mL)**		103 (4)		0.42 (8)
**ALT**_**ss**_ **(IU/mL)**	All-DAA	26.3 (9)	2.4 x 10^−7^	0.48 (8)
	TVR+PR	43.4 (6)		
**λ** **(/day)**	NS5A-containing	0.27 (8)		0.30 (25)
	NS5A-free	0.17 (8)	0.00015	
Correlation η_ALTss_, η_ALT0_				0.57 (14)
**Proportional residual errors**				0.21 (5)

RSE, residual standard error; VL, viral load; IU, international units; GT, genotype; DCV, daclatasvir; ASV, asunaprevir; 3D, paritaprevir/ritonavir, ombitasvir and dasabuvir; RBV, ribavirin; SOF, sofosbuvir; SMV, simeprevir; TVR, telaprevir; PR, pegylated interferon and ribavirin; ALT, alanine transaminase; DAA, direct-acting antivirals

^a^Infected hepatocytes are cleared with a rate δ. The free virions V are released from the infected cells at a rate ρ, infect the target cells at a rate β and are cleared from the circulation with a rate *c*. Antivirals block the production of new virus with an effectiveness ε. ALT_0_ represent baseline ALT, while ALT_ss_ represent ALT values at steady-stade, represented by week-4 value in this study; λ is the rate of ALT decline from the baseline to a new lower set point value after treatment initiation.

**Fig 2:**
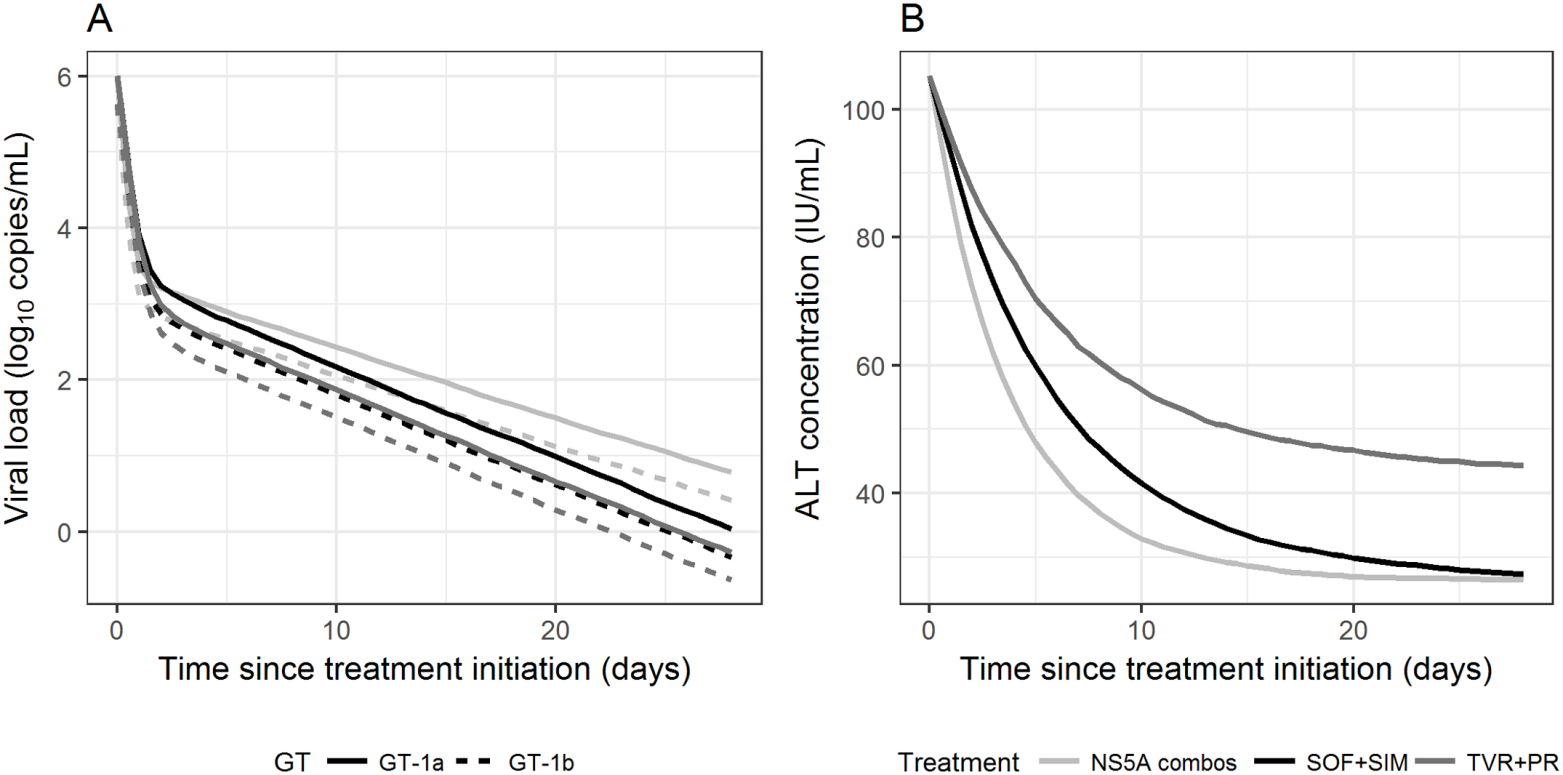
Predicted kinetic profiles obtained by simulations from the viral and ALT kinetic models. (panel **A**) Different viral kinetics according to HCV-genotypes, NS5A-inhibitors and interferon administration, (panel **B**) Different ALT kinetics according to NS5A-inhibitors and interferon administration. ALT, alanine transaminase; IFN, interferon.

The mean effectiveness of antiviral treatment (ε) was 99.8% and 99.9% in patients treated with all-DAA or TVR+PR treatments, respectively (p = 0.01). Among the tested covariates (see Methods) NS5A-inhibitors significantly affected the clearance of free-virus (c) and the loss rate of infected-cells (δ). Indeed, patients receiving a NS5A-inhibitor had a faster rate of viral-clearance (7.88 *vs*. 5.28 d^-1^ in NS5A-free; p<0.001), and a lower rate of loss of infected-cells (0.21 *vs*. 0.273 d^-1^ in NS5A-free; p = 0.0012). Thus, NS5A-inhibitors were associated with a more rapid first-phase decay of HCV-RNA, but a slower second-phase, as can be seen by the kinetics obtained by simulation in Fig 2, panel A.

### Impact of different DAA-combinations on ALT kinetics

By Cox analysis, the use of IFN and of NS5A-free regimens were associated with higher risk of not achieving normal ALT values before EOT (both p<0.0001), even after normalization for several confounding variables (Table 3).

**Table 3:** Cox analysis for factors influencing ALT normalization during treatment.

Variables	Hazard ratio of achieving normal ALT					
	Crude		Adjusted^a^		Adjusted^b^	
	HR (95% C.I.)	p-value	HR (95% C.I.)	p-value	HR (95% C.I.)	p-value
**pegIFN administration (0**^c^ **vs. 1)**	**0.34 (0.21–0.54)**	**<0.0001**	**0.29 (0.17–0.50)**	**<0.0001**	-	-
**HCV subtype (1a**^c^ **vs. 1b)**	1.39 (0.94–2.06)	0.098	1.32 (0.88–1.98)	0.176	0.89 (0.58–1.36)	0.581
**Baseline ALT**	**0.99 (0.99–0.00)**	**0.001**	**0.99 (0.99–0.99)**	**<0.0001**	**0.99 (0.99–0.99)**	**<0.0001**
**NS5A-inhibitors administration (0**^**c**^ **vs. 1)**	**3.41 (2.27–5.13)**	**<0.0001**	-	-	**3.51 (2.24–5.48)**	**<0.0001**
**HCV-RNA decay BL-2 weeks** **(per 1 log**_**10**_ **increase)**	1.27 (0.98–1.66)	0.076	1.04 (0.76–1.44)	0.796	1.14 (0.82–1.58)	0.441
**Baseline HCV-RNA (per 1 log**_**10**_ **increase)**	**0.69 (0.53–0.89)**	**0.004**	0.76 (0.54–1.07)	0.115	0.73 (0.51–1.04)	0.080
***Previous treatment experience***						
**Naïve**^c^	1	-	-	-	-	-
**Relapser**	0.85 (0.48–1.52)	0.588	-	-	-	-
**Non responder**	0.77 (0.47–1.26)	0.297	-	-	-	-
**Other**	0.83 (0.36–1.95)	0.676	-	-	-	-
**Previous PI experience (0**^c^ **vs. 1)**	1.30 (0.63–2.67)	0.482	-	-	-	-
**Favorable IL28B genotype (CC**^c^ **vs. CT/TT)**	0.66 (0.33–1.31)	0.232	-	-	-	-
**Ribavirin co-administration**	**0.58 (0.37–0.93)**	**0.025**	1.06 (0.65–1.74)	0.822	0.71 (0.44–1.16)	0.168

^a^ Adjusted for pegIFN administration, HCV-RNA decay BL-2w, baseline HCV-RNA, ribavirin administration.

^b^ Adjusted for NS5A-inhibitors administration, HCV-RNA decay BL-2w, baseline HCV-RNA, ribavirin administration.

^c^ Reference group (dummy).

ALT, alanine transaminase; BL, baseline; CI, confidence interval; HR, hazard ratio; pegIFN, pegylated interferon; PI, protease inhibitors. Normal ALT values were considered as <55 IU/ml in men, and <45 IU/ml in women. Only factors with p<0.100 in univariate analysis were included in multivariate analysis. HR in boldface represents factors having a p-value <0.050.

Notably, by mathematical modelling the rate of ALT decline (λ) was significantly lower in NS5A-free regimens than in NS5A-containing (0.17 *vs*. 0.27 d^-1^, respectively; p<0.001), with no significant difference in TVR+PR patients and those receiving SOF+SIM (Table 2). However, on the long run, IFN-treated patients had a significantly higher ALT steady state (ALT_ss_), than those receiving IFN-free regimens (43.4 *vs*. 26.3 IU/mL; p<0.001), regardless of NS5A-inhibitors administration. Typical patterns of ALT kinetics in the different treatment groups are shown in Fig 2, panel B.

### Joint kinetics of HCV-RNA and ALT

**Fig 3:**
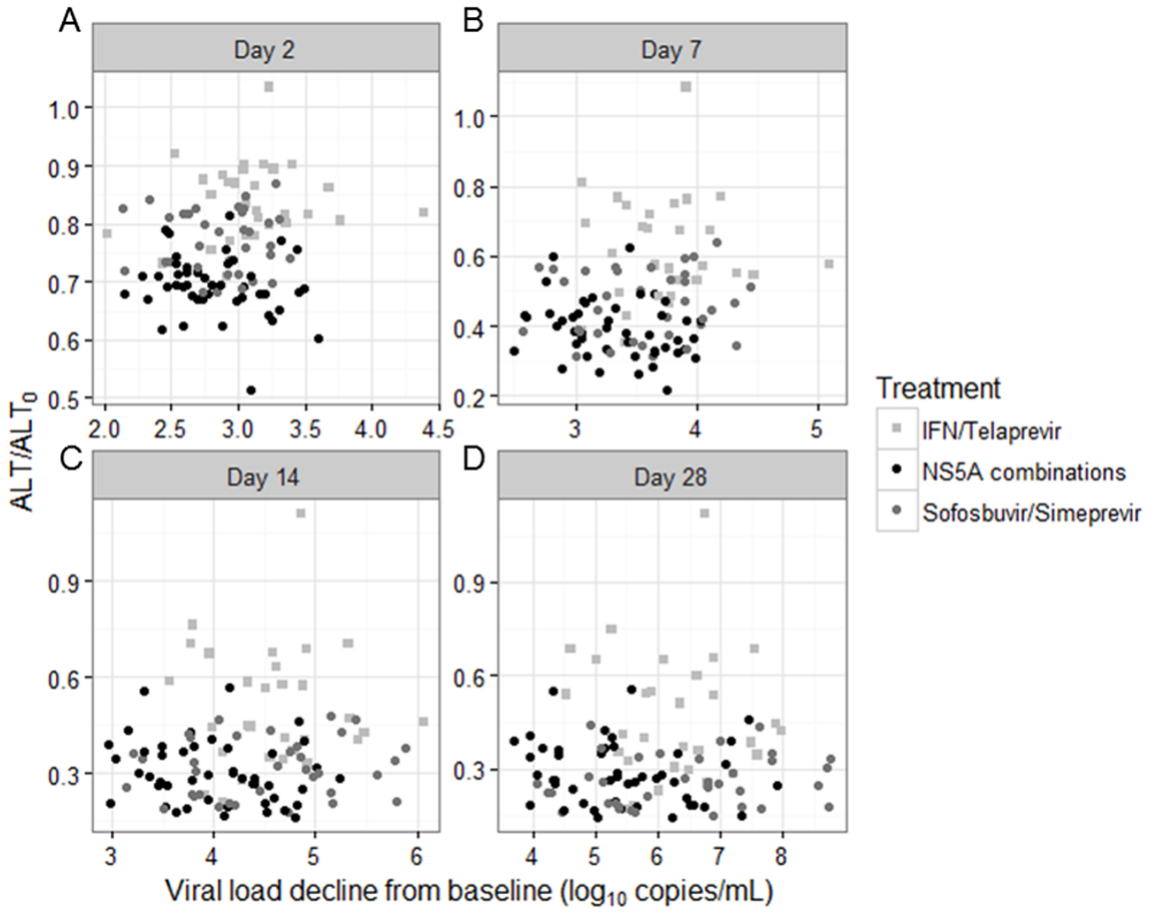
Decrease of viral load and ALT at different time points under treatment according to NS5A-inhibitors and interferon administration. These graphs shows the median of predictions of ALT and HCV-RNA in different groups of treatment obtained by simulations from the parameter estimates of the two models. ALT, alanine transaminase; IFN, interferon.

Mathematical modeling showed no association between the viral kinetic and the ALT kinetics parameters (all correlation coefficients <0.4) in the overall population, including the loss-rate of infected-cell δ and the ALT normalization rate λ (S1 and S2 Figs). This remained true when analyzing separately patients receiving IFN+TVR and all-DAA (S1 and S2 Figs). Because the kinetics of viral decline was more profound in patients treated with IFN+TVR while the ALT decline was slower, it is not surprising to find that, for a given level of viral-load reduction, the corresponding level of ALT decline is much larger in patients treated with IFN-free regimen than in patients treated with IFN+TVR (Fig 2, panels A and B). This is also illustrated in Fig 3, where the predicted levels of ALT and HCV-RNA at different time-points are represented. For none of these time-points, the ALT decline levels were found correlated with HCV-RNA decline, even when data were analyzed separately for each treatment group (not shown). This, in other words, suggests that the decline of ALT and HCV-RNA are not reflecting similar mechanisms, and that these mechanisms may be different in IFN-treated and all-DAA.

## Discussion

To our knowledge, this is the first study analyzing and comparing the different kinetics of HCV-RNA and ALT during all-DAA treatments, unveiling the effect of a specific DAA-class. We focused our study on GT-1 patients with cirrhosis, a critical population of patients with peculiar physio-pathological and anatomical characteristics, poorly studied for viral kinetics.

Our mathematical inference predicted a markedly faster and sharper ALT kinetic during all-DAA treatment than with TVR+PR, as well as a lower ALT steady-state at week-4.

In the context of IFN-based therapy, modeling papers tend to support a correlation between HCV-RNA and ALT kinetics [4], though with large inter-patients variability according to liver status [12]. This correlation was explained by IFN use, that acts by establish a non-virus-specific antiviral state[15], ultimately leading to a progressive clearance of serum HCV-RNA mediated by both clearance of circulating virions (first-phase) and, afterwards, by the death or clearance of productively-infected hepatocytes (second-phase)[1], eventually leading to serum ALT increase. Indeed, previous kinetics studies in IFN-treated patients showed a positive correlation between lower infected-cell death (δ) and persistently normal ALT values [16].

The typical ALT decay expected in patients with elevated ALT during effective IFN-treatment would thus be a superposition of two trends: 1) an early ALT decline due to initial viral clearance; and 2) a slower increase during the first 4–8 treatment weeks due to IFN-activity, 3) reversible after EOT in sustained virologic responders. We indeed observed this fluctuating ALT decay during TVR+PR treatment, when the initial ALT decay was followed by possible re-alteration and, lastly, by ALT normalization after EOT.

Notably, after achieving SVR, 73.1% of TVR+PR patients had normal ALT values, a significantly lower proportion respecting to all-DAAs treated patients (92%), but still higher of the 67% observed in cirrhotic patients receiving only IFN[12], supporting a further activity of TVR decay and ALT normalization. Indeed, recently DAAs, and TVR *in primis*, were proposed by kinetic studies to act by progressive reduction of intracellular viral-RNA down to its disappearance (“cell-cure”), rather than by inducing cell-death[7, 8]. The results of our study support this hypothesis, by demonstrating a biphasic ALT decline in which only ALT decline and normalization were observed: “healthy” ALT levels were always achieved during all-DAA treatment before complete HCV-RNA clearance, and then maintained until EOT.

The positive effects of all-DAA regimens on ALT kinetics was not simply an effect of a more profound HCV-RNA decay, and, consequently, of a sharper reduction in HCV-related pro-inflammatory status[17]. Indeed, the improved ALT decline at each analyzed time point during all-DAA treatments was confirmed also after normalization for HCV-RNA decay, again supporting an independent process of cell-cure by DAAs.

By analyzing different all-DAA combinations, we demonstrated, for the first time to our knowledge, a significant enhancement of ALT declines by NS5A-inhibitors. The use of this DAA-class also improved the concomitant first-phase of viral kinetics, in agreement with previous studies that demonstrated a rapid inhibition of viral assembly and secretion by NS5A-inhibitors[6]. NS5A-inhibitors may thus simultaneously potentiate early HCV-RNA decay by accelerating viral clearance rate from serum [6], and “anticipate” the process of cell-cure by more rapid intracellular inhibition of HCV life-cycle, even though this last hypothesis needs to be confirmed by *in vitro* studies, not yet available. Notably, both these positive effects were lost during second-phase, when δ was low and the predicted ALT kinetic profile was no longer NS5A-dependent.NS5A-inhibitors are now a universal backbone for first-line DAA regimens [18, 19], and their proposed ability in improving cell-cure further supports their preferential use as initial treatment for chronic or acute HCV infection, along with their high antiviral activity and safety profile.

Our study has some points of potential weakness. It does not include a control-population of patients receiving only PR, though TVR has a poor effect on vRNA kinetics[20], and data on “pure IFN” treatments are widely available in literature as historical controls. In addition, ALT values at early time points, such as 48h and week-1 of treatment, were available only for few TVR+PR treated patients, making the Cox analysis less precise during the first days.

Another point worth to be considered is that we analyzed a population of cirrhotic patients, in whom liver architecture is altered, and both HCV-RNA and ALT decays can be affected[12, 21, 22]. Even if our results and conclusions may be not directly extended to non-cirrhotic patients, the presence of cirrhosis seemed not to affect glaringly ALT decline. In a recent study with SOF+ledipasvir+GS9669/GS-9451, including mostly non-cirrhotic patients, ALT normalization was achieved in 90% of patients by day-14 [14], *vs*. 85% of our all-DAA patients. Differently from ALT, viral kinetics was generally reduced in our study. Our free-virus clearance rate value is lower than previous estimates (c<8 *vs*. c = 22.3 day^-1^)[6], maybe as consequence of advances cirrhosis and/or few early sampling-points. Second-phase was also quite slow and associated with poor response rates at week-4, concordantly with previous results in cirrhotic patients[23]. Whether the absence of correlation between second phase of viral decline and the normalization rate of ALT also holds in non-cirrhotic patients will need to be examined.

Overall treatment effectiveness ε was 0.998, matching previous results obtained in non-cirrhotic patients[7, 24, 25]. However, viral-kinetic response under treatment is not necessarily correlated with treatment outcome[26], and indeed TVR+PR patients achieved SVR_12_ only in 66.7% of cases, *vs*. 93.8% in all-DAAs. In the latter population, SVR_12_ rates were reduced in CTP-B patients (88.9%) compared to CTP-A (95.6%), concordant with previous results from the SOLAR study [27], and more recent data on SOF/DCV in ALLY-1 trial [28], and SOF/velpatasvir combination [29]. These results probably depend upon the severe liver alteration in decompensated patients, that deeply affect blood perfusion and metabolic capacity of the residual liver-tissue, making “cell cure” by DAAs much more difficult. Indeed, in our CTP-B patients also ALT kinetics was particularly slow, and 12.5% of CTP-B patients reached EOT with still altered ALT levels *vs*. only 1.6% of CTP-A patients; p = 0.08.

In summary, we provided a detailed analysis of HCV-RNA and ALT kinetics profiles in cirrhotic GT-1 patients treated with different DAAs-based regimens, with particular attention to the effect of NS5A-inhibitors use and highlighting differences with previous IFN-containing regimens. Our results uncovered a strikingly fast ALT normalization in patients receiving NS5A-inhibitors, in contrast with a persistent ALT alteration in patients receiving IFN, independent by HCV-RNA clearance. Considering serum ALT levels as a surrogate parameters of hepatocyte turnover [30, 31], the DAA-driven “sparing” and curing of liver cells (differently by the cytopathic effect of IFN) may provide an intriguing rationale for these results.

## Supporting information

S1 Supplementary Material (DOCX)

S1 Fig Correlation between individual random effect of the viral kinetic and ALT kinetic model in IFN-treated patients. (TIFF)

S2 Fig Correlation between individual random effect of the viral kinetic and ALT kinetic model in IFN free (all DAAs) treated patients. (TIFF)
