# Supplementary material for: Improvement of ALT decay kinetics by all-oral HCV treatment: Role of NS5A inhibitors and differences with IFN-based regimens
Source: PLoS One. 2017 May 18;12(5):e0177352. doi: 10.1371/journal.pone.0177352 (PMC5436665; doi:10.1371/journal.pone.0177352)
Supplement: S1 Supplementary Material — (DOCX) [file pone.0177352.s001.docx]

**S1 SUPPLEMENTARY MATERIAL**

**Material and Methods**

**Study population**

All patients had baseline and week-4 HCV-RNA and ALT values available. In addition, 26/111 (23.4%) of patients had available early HCV-RNA quantification at 4h; 44/111 (39.6%) at 6-8h; 49/111 (44.1%) at 24h; 109/111 (98.2%) at 48h; 18/111 (16.2%) at 72h; 80/111 (72.1%) at week-1; and 111/111 (100%) at week-2. Regarding ALT, 14/111 (12.6%) patients had early ALT values available at 8h; 23/111 (20.7%) at 24h; 41/111 (36.9%) at 48h; 72/111 (64.9%) at week-1; and 111/111 (100%) at week-2.

**HCV-RNA and ALT quantification**

HCV-RNA quantification was performed using Abbott RealTime HCV assay (Abbott Laboratories, Abbott Park, Illinois, U.S.A.), with a lower limit of detection (LLOD) and quantification (LLOQ) of 12 IU/ml; or with COBAS® AmpliPrep/COBAS® TaqMan® HCV Qualitative Test, v2.0 (LLOD=LLOQ=15 IU/ml; Roche Molecular Systems Inc.).

ALT were quantified by Dimension Vista^®^ 500 System (Siemens Healthcare Diagnostics Inc., Newark, DE U.S.A.).

**Mathematical modeling of early HCV-RNA and ALT kinetics**

For HCV kinetics, we used the standard biphasic model, assuming a constant number of target-cells. The viral kinetics model considers two populations of hepatocytes, the target-cells, T, and the infected-cells, I. Here, as only short-term data were considered, we assumed that the number of uninfected hepatocytes remained constant (T=T_0_) during the study period. Infected hepatocytes are cleared with a rate δ. The free virions V are released from the infected cells at a rate p, infect the target cells at a rate β and are cleared from the circulation with a rate c. In this model, antivirals block the production of new virus with an effectiveness ε.

$$\frac{dI}{dt}=\beta VT_{0}-\delta I$$

$$\frac{dV}{dt}=\left( 1-\varepsilon\right)pI-cV$$

For ALT kinetics, we used an exponential model which explains the decrease of ALT from the higher baseline ALT_0_ (before treatment initiation) to a lower baseline ALT_ss_.

$$ALT\left( t \right)=\left( {ALT}_{0}-{ALT}_{ss} \right)\exp\left( -\lambda t \right)+{ALT}_{ss}$$

where λ is the rate of ALT decline from the baseline to a new lower set point value after treatment initiation.

The effects of the following covariates were tested: NS5A inhibitors use, protease inhibitors use, IFN administration, RBV administration, gender, HCV-subtype (1a vs 1b), previous treatment experience (naïve, no response [including adverse events, breakthrough and non-responders], relapsers) and analytic technique for HCV-RNA quantification (see above). We tested covariate effect on univariate analysis on each kinetic parameter using likelihood ratio test. Only covariate effects that appeared significant in univariate analysis were tested in the multivariate analysis.
