# Supplementary figures and images for: Improvement of ALT decay kinetics by all-oral HCV treatment: Role of NS5A inhibitors and differences with IFN-based regimens
Source: PLoS One. 2017 May 18;12(5):e0177352. doi: 10.1371/journal.pone.0177352 (PMC5436665; doi:10.1371/journal.pone.0177352)

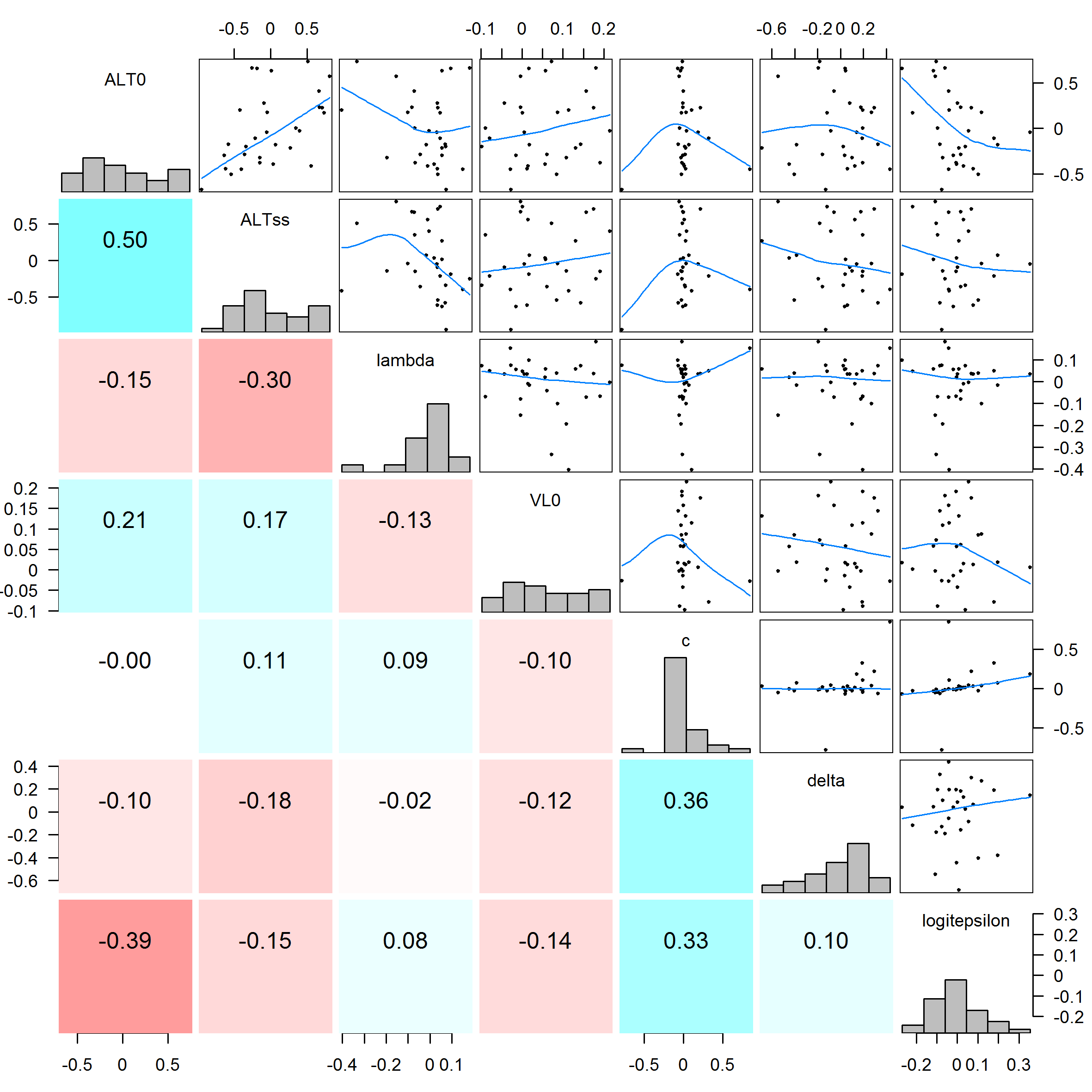

Supplement: S1 Fig — (TIFF) [file pone.0177352.s002.tiff]

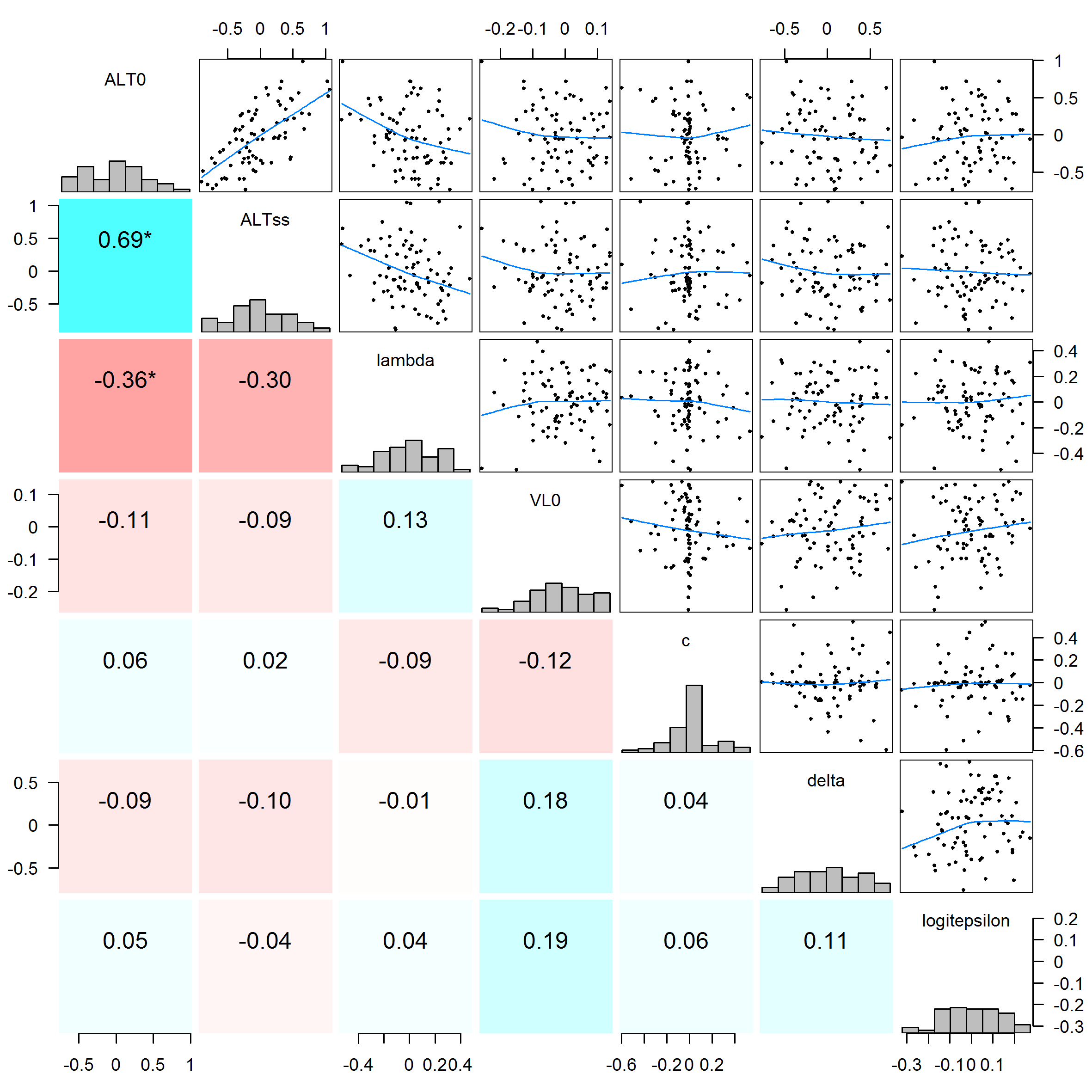

Supplement: S2 Fig — (TIFF) [file pone.0177352.s003.tiff]
